# An Ant Colony Optimization Based on Information Entropy for Constraint Satisfaction Problems

**DOI:** 10.3390/e21080766

**Published:** 2019-08-06

**Authors:** Boxin Guan, Yuhai Zhao, Yuan Li

**Affiliations:** 1 School of Computer Science and Engineering, Northeastern University, Shenyang 110819, China; 2 School of Information Science and Technology, North China University of Technology, Beijing 100144, China

**Keywords:** ant colony optimization, constraint satisfaction problem, information entropy, local search

## Abstract

Solving the constraint satisfaction problem (CSP) is to find an assignment of values to variables that satisfies a set of constraints. Ant colony optimization (ACO) is an efficient algorithm for solving CSPs. However, the existing ACO-based algorithms suffer from the constructed assignment with high cost. To improve the solution quality of ACO for solving CSPs, an ant colony optimization based on information entropy (ACOE) is proposed in this paper. The proposed algorithm can automatically call a crossover-based local search according to real-time information entropy. We first describe ACOE for solving CSPs and show how it constructs assignments. Then, we use a ranking-based strategy to update the pheromone, which weights the pheromone according to the rank of these ants. Furthermore, we introduce the crossover-based local search that uses a crossover operation to optimize the current best assignment. Finally, we compare ACOE with seven algorithms on binary CSPs. The experimental results revealed that our method outperformed the other compared algorithms in terms of the cost comparison, data distribution, convergence performance, and hypothesis test.

## 1. Introduction

The constraint satisfaction problem (CSP) is an assignment that consists of a set of variables that satisfy some constraints [1,2,3,4]. CSP can be solved by assigning specific values to variables in accordance with the constraint conditions [5,6,7,8]. The problem has been applied in a multitude of domains in real life, such as scheduling [9,10], task planning [11,12], gate assignment [13,14], and the reserve design problem [15,16].

To solve the CSP, complete methods based on the backtracking mechanism [17,18] explore all possible solutions until they find a feasible solution or prove the non-existence of any solution at all. These complete methods are often integrated with filtering technologies, which are effective in the reduction of the domains. Although the completeness appears to be an ideal property, it is difficult to solve high complex CSPs.

As a result, incomplete methods that include pure random walk (PRW) algorithms [5,19] and evolutionary optimization algorithms [20] have been proposed to find the approximately optimal solution in an opportunistic way. The incomplete methods tend to randomly explore the space and follow heuristic means to search for the most promising domains. The efficiency of the PRW algorithms has been already proved by the in-depth studies on some applications of CSPs. However, they could not ensure the quality of solutions as usual. On the premise of guaranteeing the quality of solutions, evolutionary optimization algorithms can greatly improve the search speed.

In recent years, evolutionary optimization algorithms have attracted attention for solving the CSP. EEMDE is a hybrid meta-heuristic differential evolution (DE) algorithm with an element exchange mechanism, and the mechanism uses the individual differential direction of moving length to maintain the population diversity [21]. PS is a discrete particle swarm optimization (PSO) algorithm that uses information about the conflicts between the variables to calculate the velocity of the individual particles [22]. GSABC is a hybrid algorithm combining artificial bee colony (ABC) and greedy local search technology [23]. ACOS [24], ACOD [24], ACON [25] and ACOU [26] are ACO-based algorithms for tackling the problem. ACOS makes use of the smallest-domain-first strategy to reinforce the search process, whereas ACOD applies the dynamic-random strategy to achieve that. ACON takes advantage of a negative-feedback mechanism to make the ant swarm explore the unknown space in the optimization process. ACOU uses a strengthened pheromone updating mechanism to enhance the pheromone on the edge that has never appeared before according to the dynamic information in the optimization process. Among these evolutionary algorithms, the performance of the ACO-based algorithms is superior.

When using the ACO-based algorithms to solve the CSP, the main task is to construct a feasible assignment that does not violate any constraints. Due to a large number of constraints, the feasible assignment is very hard to find in most test cases. Thus, the ACO-based algorithms try to find an assignment with a low cost value as much as possible. Although some ACO-based algorithms have been proven to be able to solve the CSP, they are still easily trapped in a locally optimal state. In this paper, an incomplete method based on ACO is proposed to solve CSPs. The new algorithm is abbreviated as ACOE, which stands for ant colony optimization based on information entropy. The idea of ACOE is that a crossover-based local search (CLS) is automatically called according to the feedback of information entropy.

The contributions of the paper are listed as follows. (1) A ranking-based pheromone updating strategy is incorporated into the ACOE algorithm to strengthen the exploratory ability of ants. (2) An automatic adjustment mechanism based on information entropy is proposed. By using the mechanism, the proposed algorithm can perform a local search when the algorithm falls into the local optimal state. (3) A crossover-based local search is used in the ACOE algorithm. Through automatically calling the CLS, ACOE is capable of maintaining the diversity of constructed assignments, and accordingly, improve the quality of the assignments.

The remaining parts of this paper are structured as follows. Section 2 gives the definition of CSP and describes the proposed ACOE algorithm for solving the CSP. Section 3 reports and discusses the experimental results. Section 4 draws the conclusion.

## 2. Methods

### 2.1. Problem Definition

We defined a CSP to be a triple (*X*, *D*, *C*), where *X* is a finite set of variables, *D* is a function that associates each variable with its domain, and *C* is a set of constraints that restrict the values that the variables can assign at the same time. A label <*x_i_*, *v_p_*> associates variable *x_i_* with a value *v_p_* from the domain *D*(*x_i_*). An assignment *A* is a set of labels where no variables appear more than once. To solve a CSP more conveniently, we represent the CSP (*X*, *D*, *C*) as an undirected graph *G* = (*V*, *E*), where *V* is the vertex and *E* is the edge. In the constructed graph, a possible label is represented by a vertex. A path containing <*x_i_*, *v_p_*> cannot contain another label for variable *x_i_*, otherwise a constraint is violated. The cost function of an assignment *A*, represented by *cost*(*A*), is the number of violated constraints in the assignment *A*. The cost is 0 if the assignment does not violate any constraints.

**Figure 1:**
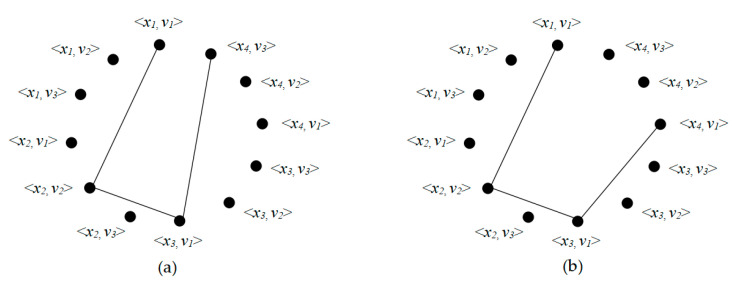
(**a**) An assignment does not violate any constraints; and (**b**) an assignment that violates a constraint.

Let us give an example of the CSP. Suppose *X* = {*x*_1,_ *x*_2,_ *x*_3,_ *x*_4_}, *D* = {*v*_1,_ *v*_2,_ *v*_3_}, and *C* = {*c*_12,_ *c*_23,_ *c*_34_} where *c*_12_ = {(*v*_1,_ *v*_2_), (*v*_2,_ *v*_3_)}, *c*_23_ = {(*v*_3,_ *v*_2_), (*v*_2,_ *v*_1_)}, and *c*_34_ = (*v*_1,_ *v*_3_). As shown in Figure 1a, the assignment does not violate any constraints is {<*x*_1,_ *v*_1_>, <*x*_2,_ *v*_2_>, <*x*_3,_ *v*_1_>, <*x*_4,_ *v*_3_>}, and the cost value of the assignment is 0. As shown in Figure 1b, the assignment {<*x*_1,_ *v*_1_>, <*x*_2,_ *v*_2_>, <*x*_3,_ *v*_1_>, <*x*_4,_ *v*_1_>} that violates one constraint because <*x*_3,_ *v*_1_> and <*x*_4,_ *v*_1_> can not be connected, hence the cost value of the assignment is 1.

### 2.2. Original Ant Colony Optimization (ACO)

ACO, proposed by Dorigo et al. [27], solves the optimization problem by simulating the behavior of real ants finding the shortest path between the nest and the food source. The ACO algorithm has the characteristics of distributed computing, information positive feedback, and heuristic search. At present, the algorithm has achieved good results in CSPs.

In ACO, artificial ants live in a discrete world, and their movement is essentially a transition from one discrete state to another. Each artificial ant releases the pheromone after constructing an assignment, and the amount of pheromone released is directly proportional to the quality of the assignment. The probability that the assignment is selected is determined by a probability distribution formula, which is updated by pheromones, heuristic information, and weights. As the probability distribution function is updated, the better assignment will be selected by subsequent ants with a higher probability. At the same time, a small portion of the pheromone is released on each assignment, allowing the ants to try to find assignments that have not been selected before.

### 2.3. Ant Colony Optimization Based on Information Entropy (ACOE)

ACOE follows the basic ACO algorithm for solving CSPs, and the process is shown in Algorithm 1. At each iteration, ant *k* constructs an assignment *A_k_*. If the cost of *A_k_* is lower than that of the current best assignment *bestA*, *bestA* is replaced by *A_k_*; otherwise, *bestA* is unchanged. Then, the pheromone value on each vertex is updated. The optimization process is repeated until a solution is found by an ant or the maximum number of iterations *N_max_* is reached. In the following, we first described the assignment construction and the ranking-based pheromone updating. Then, we introduced the automatic adjustment mechanism based on information entropy and the crossover-based local search. Finally, we discussed parameter settings.


**Algorithm 1 ACOE**
**Input:** a *CSP* (*X*, *D*, *C*), maximum number of iterations *N_max_*, number of ants *N_ant_*
**Output:**
*bestA*
1: Initialization2: **repeat**3:   **for**
*k* = 1 to *N_ant_*
**do**4:     Construct a complete assignment *A_k_*5:     **if** cost(*A_k_*) < cost(*bestA*) **then**6:        *bestA* ← *A_k_*7:     **end if**8:     **if** the condition is satisfied **then**9:        *bestA* ← CLS(*bestA*)10:    **end if**11:  **end for**12:  Update pheromone on each vertex13: **until** cost(*bestA*) = 0 ∨ *N_max_* is reached14: **return**
*bestA*

#### 2.3.1. Assignment Construction

For constructing the assignment, each ant starts with an empty assignment and then iteratively selects the next vertex that is not assigned to the assignment. The probability of selecting the vertex of the assignment *A_k_* is defined as:

(1) $$p_{A_{k}}(<x_{i},v_{p}>)=\frac{{\left[\tau_{A_{k}}(<x_{i},v_{p}>)\right]}^{\alpha }{\left[\eta_{A_{k}}^{}(<x_{i},v_{p}>)\right]}^{\beta }}{\sum_{p=1}^{m}{\left[\tau_{A_{k}}(<x_{i},v_{p}>)\right]}^{\alpha }{\left[\eta_{A_{k}}^{}(<x_{i},v_{p}>)\right]}^{\beta }},$$

(2) $$\eta_{A_{k}}^{}(<x_{i},v_{p}>)=\frac{1}{1+\cos t((<x_{i},v_{p}>)\cup A)-\cos t(A)},$$

where *τ_Ak_*(<*x_i_*, *v_p_*>) is the pheromone value on the vertex <*x_i_*, *v_p_*>; *α* is the parameter determining the weight of the pheromone value; *η_Ak_*(<*x_i_*, *v_p_*>) is the heuristic information of selecting the vertex <*x_i_*, *v_p_*> [28], which is inversely proportional to the number of new violated constraints when assigning <*x_i_*, *v_p_*> to *A_k_*; *β* is the parameter determining the weight of the heuristic information; *m* is the number of values for each variable. The pseudo-code of the assignment constructed by ant *k* is given in Algorithm 2.


**Algorithm 2 Assignment Construction**
**Input:** ant *k*
**Output:**
*A_k_*
1: Selects a starting vertex <*x_i_*, *v_p_*>2: Place ant *k* on the vertex <*x_i_*, *v_p_*>3: *A_k_* ← <*x_i_*, *v_p_*>4: **while** |*A_k_*| < |X| **do**5:     Select vertex <*x_j_*, *v_q_*> that is not assigned to *A_k_*6:     Move ant *k* to <*x_j_*, *v_q_*>7:     *A_k_* ← *A_k_* ∪ <*x_j_*, *v_q_*>8: **end while**9: **return**
*A_k_*

#### 2.3.2. Ranking-Based Pheromone Updating

After each ant constructs a complete assignment, the pheromone values are updated. In ACOE, the ants are sorted by the costs of the constructed assignments, and the contribution of the pheromone updating is weighted according to the rank *r* of the ant. We used the weight *r* for the *r*-th best ant. Thus, the pheromone values were updated by:

(3) $$\begin{array}{l} \tau_{A_{k}}(<x_{i},v_{p}>)=(1-\rho )\tau_{A_{k}}(<x_{i},v_{p}>)+\Delta \tau_{A_{k}}(<x_{i},v_{p}>)\\ if\;\tau_{A_{k}}(<x_{i},v_{p}>)\;<\tau_{\min},\;then\;\tau_{A_{k}}(<x_{i},v_{p}>)\;\leftarrow \tau_{\min}\\ if\;\tau_{A_{k}}(<x_{i},v_{p}>)\;>\tau_{\max},\;then\;\tau_{A_{k}}(<x_{i},v_{p}>)\;\leftarrow \tau_{\max} \end{array}$$

(4) $$\Delta \tau_{A_{k}^{}}^{}(<x_{i},v_{p}>)=\left\{ \begin{array}{ll} \frac{1}{r*\cos t(A_{k}^{})} & if\;\mathrm{ant}\;k\;\mathrm{is}\;\mathrm{the}\;r-\mathrm{th}\;\mathrm{best}\;\mathrm{ant}\\ 0 & \;otherwise \end{array}\right.,$$

where *ρ* is the pheromone evaporation rate (0 < *ρ* < 1); *r* is the ranking index; and ∆*τ_Ak_*(<*x_i_*, *v_p_*>) is the increased pheromone caused by the ant *k*. If ant *k* is the *r*-th best ant, the increased pheromone on the vertex <*x_i_*, *v_p_*> belonging to the assignment *A_k_* is inversely proportional to the cost multiplied by *r*. A smaller *r* causes more pheromones to be increased on the vertices belonging to the assignment.

As indicated by (3), the range of *τ_Ak_* is between the minimum pheromone *τ*_min_ and the maximum pheromone *τ*_max_ in the condition of *τ*_min_ ≤ *τ_Ak_* ≤ *τ*_max_ (0 < *τ*_min_ ≤ *τ*_max_) [28]. Once the value of *τ_Ak_* exceeds the range, the value will change to the nearest end-point.

The degree to which the global information is contributed depends on the quality of the generated assignments. A better assignment is more likely to make a greater contribution to the future assignments. The pheromone updating strategy based on ant ranking make assignments with lower costs more contribution to the global optimization. Thus, the global search ability of ACOE is enhanced.

#### 2.3.3. Automatic Adjustment Mechanism Based on Information Entropy

Information entropy is used to measure the expected value of a random variable. The larger the information entropy of a variable, the greater its uncertainty, that is, more information is needed to determine this variable. The information entropy of an assignment is the sum of the information entropy of all variables:

(5) $$H(A_{k})=-\sum_{i=1}^{n}\sum_{p=1}^{m}p_{A_{k}}(<x_{i},v_{p}>)\log p_{A_{k}}(<x_{i},v_{p}>),$$

where *p_Ak_*(<*x_i_*, *v_p_*>) is the probability that the vertex <*x_i_*, *v_p_*> is selected in the assignment *A_k_*; *n* is the number of variable; *m* is the number of value; H(*A_k_*) is the information entropy of the assignment constructed by ant *k*; and the logarithm takes 2 as the base. ACOE solves the CSP by constantly comparing the current global best assignment and the best assignment in the current iteration. The comparison process for the two assignments is defined in the formula below:

(6) $$|H(bestA)-H(best^{t}A)|<\theta ,$$

where H(*BestA*) is the current global best assignment; H(*Best^t^A*) is the best assignment in the *t*th iteration; and *θ* is the specified switch parameter.

At the beginning of ACOE, the pheromones on each vertex are equal and the information entropy is the largest. As the number of iterations increases, the pheromones on the vertices that found by ants increase, whereas the pheromones on the other vertices decrease. At the same time, the changing process of these pheromones leads to a reduction in the information entropy of each assignment. When the difference between H(*BestA*) and H(*Best^t^A*) is very small, the proposed algorithm performs a local search (see Section 2.3.4).

#### 2.3.4. Crossover-Based Local Search

To enhance the search ability of ACOE, we incorporated a local search (LS) into the proposed algorithm. The LS uses a crossover operation to optimize the current best assignment. Thus, this LS is called CLS. For solving CSPs, assignments with lower costs are generally more inclined to be selected by ants. Therefore, the excellent assignments with lower costs are selected to explore its neighborhood by using the CLS procedure, and better assignments are expected to be obtained. In ACOE, a crossover operation is performed if the difference of the information entropy between the current global best solution and the best solution in the *t^th^* iteration is less than *θ*. The current best assignment and other randomly selected assignments will be crossed to obtain a new assignment. Suppose the current best assignment is *bestA* = {*<x*_1_, *v*_1_>, <*x*_2_, *v*_2_>, <*x*_3_, *v*_3_>, …, <*x*_n−1_, *v_n_*_−1_>, <*x_n_*, *v_n_*>}, where *n* is the number of variables; the randomly selected assignment is {<*x*_1_, *v*_1_>, <*x*_2_, *v*_3_>, <*x*_3_, *v*_4_>, …, <*x_n_*_−1_, *v_n_*_−2_>, <*x_n_*, *v_n_*_−3_>}. We selected a random integer uniformly distributed between 1 and (*n*−1) as the crossover point, and we assumed 2 was the intersection point in this example. Then, *bestA* and *A_u_* crossed to generate a new assignment *C* = {<*x*_1_, *v*_1_>, <*x*_2_, *v*_2_>, <*x*_3_, *v*_4_>, …, <*x_n_*_−1_, *v_n_*_−2_>, <*x_n_*, *v_n_*_−3_>}. If the newly obtained assignment has a lower cost value than the best assignment, the new assignment will replace the best assignment. Otherwise, the best assignment will be preserved. The pseudo-code of CLS is shown in Algorithm 3.


**Algorithm 3 CLS**
**Input:** *bestA*, number of crossover operations *L*, number of values *m***Output:** *bestA*1: **for**
*u* = 1 to *L*
**do**2:      *A_u_* ← select a random assignment3:      crossover point ← U [1, *m* − 1]4:      *C* ← Crossover(*bestA*, *A_u_*)5:      **if**
*cost*(*C*) < *cost*(*bestA*) **then**6:           *bestA* ← *C*7:      **end if**8: **end for**9: **return**
*bestA*

#### 2.3.5. Parameter Setting

ACOE has some parameters: The number of ants *N_ant_*, the minimum pheromone *τ_min_*, the maximum pheromone *τ_max_*, the specified switch parameter *θ*, the pheromone evaporation rate *ρ*, and the weight parameters *α* and *β*. We briefly analyzed the impact of these parameters on this proposed algorithm. *N_ant_* was set to 10: The running time will increase if *N_ant_* has a larger value; the cost will increase if *N_ant_* has a smaller value. *τ_min_* was set to 0.01 and *τ_max_* was set to = 4 according to previous studies [24,29]. *θ* was set to 0.01: If *θ* has a smaller value, CLS can hardly work; if *θ* has a larger value, the running time will increase due to calling CLS multiple times. *β*, *α,* and *ρ* have an impact on the exploratory behavior of ants. *β* was set to 10, *α* was set to 2, and *ρ* was set to 0.01. ACOE was run 30 times on the same test case (Test 7) with different combinations of *β*, *α*, and *ρ*. Then, the lowest cost value corresponding to a combination of the three parameters was recorded. The details of the experimental results are shown in Table 1. In the table, *β* was set to 6, 8, and 10; *α* was set to 2, 3, 4, and 5; *ρ* was set to 0.01, 0.02, 0.03, 0.04, and 0.05. The other values represent the lowest costs obtained by ACOE with different *β*, *α*, and *ρ*.

## 3. Results and Discussion

### 3.1. Datasets

In the paper, four classes of binary CSP test cases were generated (Class 1, Class 2, Class 3, and Class 4), and each class contained six specific test cases. The generated test cases were represented by four components *< n, m, p*_1_, *p*_2_ >, where *n* is the number of variables, *m* is the domain for each variable, *p*_1_ is the connectivity of the constraint graph, and *p*_2_ is the tightness of the constraints. Furthermore, the constrainedness of a generated test case can be defined by the *k*-value (the range is 0 to 1), and the *k*-value can be calculated according to Equation (7) [30,31]. A CSP is under-constrained and can be solved when *k* is less than 1, whereas a CSP is over-constrained and usually difficult to solve when *k* is greater than 1. More details of the generated test cases are shown in Table 2.

(7) $$k(n,m,p_{1},p_{2})=\frac{n-1}{2}p_{1}\log_{m}(\frac{1}{1-p_{2}}).$$

### 3.2. Cost Comparison

The cost value is an important index to evaluate the performance of the compared algorithms. For each test case, we ran eight algorithms (ACOE, ACOS, ACOD, ACON, ACOU, EEMDE, PS, and GSABC) 30 times respectively. The minimum cost (Min), the average cost (Avg), and the maximum cost (Max) were recorded, and the experimental results are given in Table 3. It can be seen from the table that the minimum cost, the average cost, and the maximum cost increase gradually increased with the growth of the *k*-value. For the small-scale problems with 100 variables (Test 1–20), the minimum cost values obtained by ACOE were not obviously superior to those obtained by the other compared algorithms. The proposed algorithm was not as good as ACON on Test 18, and it was inferior to ACON and ACOU on Test 20. All the average cost values found by ACOE were the lowest, whereas the maximum cost values obtained by this proposed algorithm were the lowest except for Test 18. For the large–scale problems with 150 variables (Test 21–40), ACOE presents more obvious advantages than the other seven compared algorithms. For the 20 test cases, ACOE gets all the best minimum costs, average costs, and maximum costs.

### 3.3. Result Distribution Analysis

**Figure 2:**
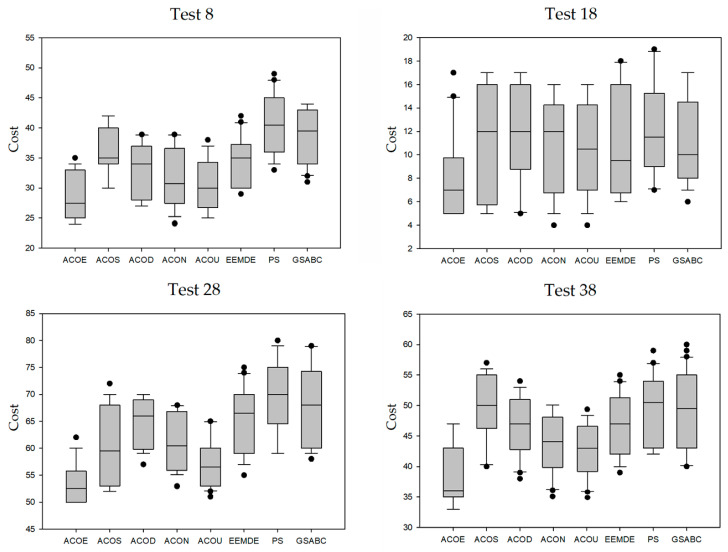
Box plots of five compared algorithms.

In this section, we conducted an analysis about the result distribution of the eight compared algorithms. Test 8, Test 18, Test 28, and Test 38 were selected as the representative of each case. For each representative, all of the cost values obtained by each compared algorithm in 30 runs were used as experimental data. We calculated the minimum point, the first quartile, the median, the third quartile, and the maximum point of the cost values of each test case, and then we used the five statistical quantities to draw the box plots (Figure 2). The median was used to describe the concentration of the experimental data, regardless of the maximum or minimum value of the data distribution. As can be seen from these box plots, the median cost of ACOE was lower than that of the other compared algorithms. In addition, the length of the box also reflected the concentration of the data. The box length of ACOE was relatively short on the four test cases. The above analysis indicates that the result distribution of the proposed algorithm was the most concentrated.

### 3.4. Convergence Analysis

**Figure 3:**
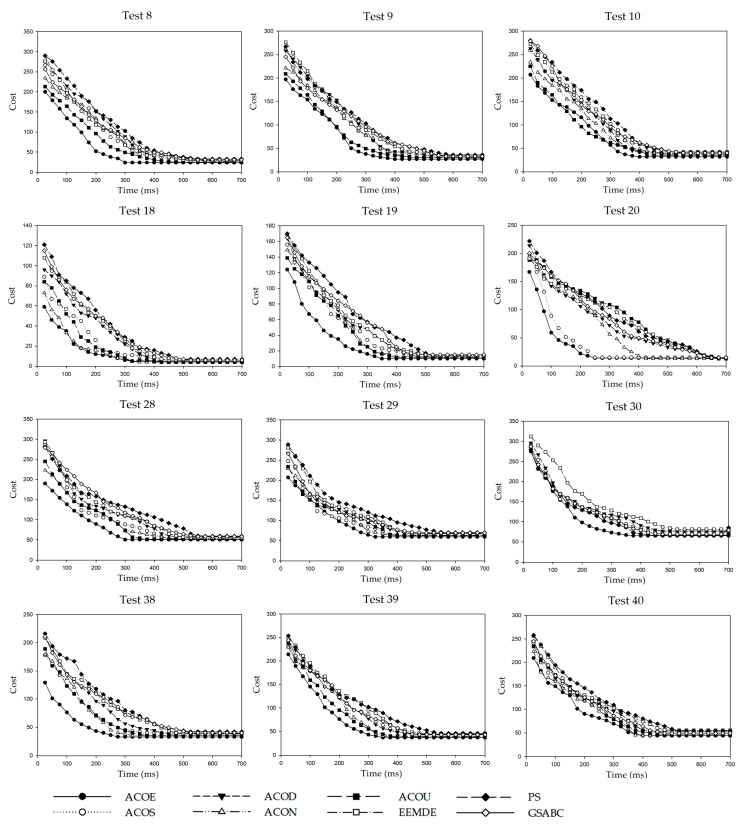
Convergence diagrams of eight compared algorithms.

Convergence means that the objective cost value evaluated by an algorithm tends to be stable after several iterations. We compared the convergence of the eight compared algorithms on Test 8, Test 9, Test 10, Test 18, Test 19, Test 20, Test 28, Test 29, Test 30, Test 38, Test 39, and Test 40, and the convergence diagrams are displayed in Figure 3. The running time (millisecond) was 100, 200, 300, 400, 500, 600, and 700, respectively, and these values were served as the scale units of the horizontal axis. The cost of each algorithm under different scale units was recorded in these diagrams. For Test 8, ACOE converged in around 300 ms, ACOU converged in about 400 ms, and the rest of algorithms converged after approximately 600 ms. For Test 20, ACOE converged only after about 250 ms, which was significantly faster than the other algorithms. For Test 28, Test 29, and Test 38, ACOE converged after approximately 300 ms, and the other compared algorithms converge between 350 ms and 550 ms. For Test 9, Test 10, Test 19, Test 30, Test 39, and Test 40, ACOE converged after approximately 400 ms, and the other compared algorithms converged after 450 ms. For Test 18, all the five algorithms were in approximately 450 ms.

### 3.5. Hypothesis Test

For the purpose of proving the validity of ACOE in coping with CSPs, we used the fisher-indep hypothesis test with a 0.05 confidence level. Thus, a significant difference could be reflected between two algorithms when the *p*-value was below 0.05. All the cost values obtained by each compared algorithm in 30 runs were collected as experimental data. The comparative results of ACOE with ACOS, ACOD, ACON, ACOU, EEMDE, PS, and GSABC are shown in Table 4. For some small–scale test cases, such as Test 1 and Test 2, ACOE was not significantly different from the other seven compared algorithms. On Test 3 and Test 11, the proposed algorithm was only significantly different from PS. ACOE performed significantly than ACOS and PS on Test 4, and it performed significantly than EEMDE, PS, and GSABC on Test 12, Test 18, and Test 20. On Test 6 and Test 13, the proposed algorithm did not perform significantly better than ACON and ACOU. The proposed algorithm was not significantly better than ACOD and ACOU on Test 7, and it was not significantly better than ACON on Test 9 and Test 19. On the large-scale test cases (Test 21–40), the *p*-values were less than 0.05 when ACOE was compared to ACOS, ACOD, ACON, ACOU, EEMDE, PS, and GSABC, which indicates ACOE was significantly better than the other algorithms.

## 4. Conclusions

CSP, as a topic of artificial intelligence, plays an important role in many real-life applications. In the paper, the ACOE algorithm was proposed to deal with the problem. On the generated CSP test cases, the performance of ACOE was evaluated from the aspects of cost comparison, data distribution, convergence performance, and hypothesis test. The results showed that the proposed algorithm had advantages in efficiency and effectiveness. However, there were limitations about the proposed algorithm on the next two aspects. First of all, although we had introduced some measurements to evaluate the performance of different algorithms, it is worth mentioning here that other evaluation measures like running time and standard deviation could be also be applied for a wider range of performance analysis. Secondly, although ACOE was evaluated on 40 test cases, the algorithm was not tested on real datasets. In the future, we will focus on the application of the proposed algorithm on real data.

## Figures and Tables

**Table 1:** Effects of *β*, *α*, and *ρ* with different settings on Test 7.

*ρ*	*β*	6				8				10			
	*α*	2	3	4	5	2	3	4	5	2	3	4	5
0.01		28	26	26	28	25	25	26	27	24	25	25	27
0.02		29	30	29	29	26	26	26	28	25	26	25	27
0.03		30	31	30	29	26	27	26	27	25	26	26	28
0.04		29	30	30	31	28	27	28	29	25	26	27	28
0.05		30	29	31	30	27	29	30	31	26	28	30	29

**Table 2:** Details of generated test cases.

	Component Set	Test Case	*p*_2_	*k*
Class 1	(100, 4, 0.14, *p*_2_)	Test 1	0.10	0.527
		Test 2	0.12	0.639
		Test 3	0.14	0.754
		Test 4	0.16	0.872
		Test 5	0.18	0.992
		Test 6	0.20	1.115
		Test 7	0.22	1.242
		Test 8	0.24	1.372
		Test 9	0.26	1.505
		Test 10	0.28	1.642
Class 2	(100, 8, 0.14, *p*_2_)	Test 11	0.12	0.426
		Test 12	0.14	0.503
		Test 13	0.16	0.581
		Test 14	0.18	0.661
		Test 15	0.20	0.743
		Test 16	0.22	0.828
		Test 17	0.24	0.914
		Test 18	0.26	1.003
		Test 19	0.28	1.094
		Test 20	0.30	1.188
Class 3	(150, 4, 0.14, *p*_2_)	Test 21	0.06	0.466
		Test 22	0.08	0.627
		Test 23	0.10	0.793
		Test 24	0.12	0.961
		Test 25	0.14	1.134
		Test 26	0.16	1.311
		Test 27	0.18	1.493
		Test 28	0.20	1.679
		Test 29	0.22	1.869
		Test 30	0.24	2.605
Class 4	(150, 8, 0.14, *p*_2_)	Test 31	0.10	0.528
		Test 32	0.12	0.641
		Test 33	0.14	0.756
		Test 34	0.16	0.874
		Test 35	0.18	0.995
		Test 36	0.20	1.119
		Test 37	0.22	1.246
		Test 38	0.24	1.376
		Test 39	0.26	1.510
		Test 40	0.28	1.648

**Table 3:** Results of the minimum cost, average cost, and maximum cost.

	Minimum Cost/Average Cost/Maximum Cost							
Test Case	ACOE	ACOS	ACOD	ACON	ACOU	EEMDE	PS	GSABC
Test 1	0/0/0	0/0/1	0/1/1	0/0/1	0/0/0	0/1/2	0/1/1	0/0/1
Test 2	0/0/1	0/1/2	0/1/2	0/0/1	0/1/1	0/0/1	0/1/2	0/1/2
Test 3	0/0/1	0/1/4	0/1/2	0/1/2	0/1/2	0/2/3	1/2/4	1/1/3
Test 4	0/0/2	0/2/5	0/1/3	0/1/3	0/0/2	0/2/3	0/2/4	0/1/2
Test 5	0/0/1	0/1/3	0/1/3	0/0/2	0/1/2	0/1/2	1/2/3	0/1/2
Test 6	0/2/4	0/4/6	0/5/8	0/3/5	0/3/4	0/4/7	1/4/6	1/3/5
Test 7	24/30/38	29/35/42	29/34/39	27/35/40	25/32/39	28/37/45	30/38/46	27/36/41
Test 8	24/28/35	30/37/42	27/32/39	24/33/39	25/31/38	29/36/42	33/40/49	31/38/44
Test 9	27/34/41	31/40/47	33/42/47	29/36/43	30/36/46	34/42/48	36/42/50	33/41/46
Test 10	32/39/45	39/48/54	40/48/47	37/43/49	35/39/48	42/50/57	42/52/59	40/46/53
Test 11	0/1/1	0/1/3	0/1/2	0/1/2	0/1/2	0/1/3	0/2/4	0/1/2
Test 12	0/2/4	1/3/5	0/3/5	0/3/4	0/2/5	1/3/6	2/4/7	0/3/6
Test 13	0/4/6	1/5/7	1/5/8	0/5/7	0/4/7	1/4/8	1/5/9	1/5/8
Test 14	1/4/7	2/6/9	2/8/10	1/4/8	1/5/10	2/7/11	3/8/12	2/8/11
Test 15	0/5/7	1/6/10	0/5/10	0/5/9	0/5/8	1/6/10	1/6/11	1/5/10
Test 16	0/6/9	2/8/10	2/9/12	1/8/12	1/7/12	3/9/13	3/10/14	2/9/13
Test 17	3/8/14	5/10/18	4/10/16	4/9/15	4/10/15	5/11/16	6/12/19	5/11/18
Test 18	5/8/17	5/11/17	5/10/17	4/10/16	5/9/16	6/9/18	7/11/19	6/10/17
Test 19	10/15/24	14/19/25	12/18/25	13/17/24	11/17/25	14/19/27	15/19/29	15/18/26
Test 20	14/18/24	15/20/27	14/21/29	13/20/28	13/19/27	16/21/31	17/23/32	17/21/30
Test 21	3/4/7	4/7/10	5/7/9	3/5/7	3/5/8	5/8/10	6/10/14	6/8/11
Test 22	5/6/11	7/9/12	7/10/14	6/9/14	7/10/13	9/13/17	9/13/19	8/12/16
Test 23	6/8/13	7/11/15	7/10/15	6/10/14	6/11/14	8/12/17	8/14/19	7/12/16
Test 24	6/9/13	8/12/16	7/12/16	8/12/15	7/11/15	9/13/18	11/15/20	8/14/19
Test 25	5/8/14	6/12/16	5/11/16	5/10/15	6/11/16	8/13/18	10/15/19	6/13/17
Test 26	24/33/41	28/40/45	27/39/45	26/38/44	27/39/42	31/42/49	33/45/52	29/41/48
Test 27	53/57/63	57/65/73	59/64/72	56/62/70	56/61/72	60/68/78	65/74/85	61/70/83
Test 28	50/52/62	52/65/72	57/64/70	53/60/68	51/59/65	55/66/75	59/69/80	58/67/79
Test 29	59/69/77	64/75/87	68/77/88	63/70/80	62/71/83	67/78/90	70/82/95	69/80/92
Test 30	65/73/84	75/83/94	77/89/95	66/75/87	69/76/89	81/95/105	85/98/105	79/92/98
Test 31	0/0/0	0/0/2	0/1/2	0/0/1	0/0/2	0/1/3	0/1/2	0/1/3
Test 32	0/1/2	2/4/5	1/3/4	0/1/3	0/2/3	2/4/6	3/5/6	2/3/5
Test 33	0/2/4	2/4/7	2/4/6	1/3/4	2/3/5	2/5/8	3/6/8	2/3/6
Test 34	1/3/6	2/5/8	2/5/8	2/4/7	2/4/8	3/5/9	3/6/10	2/5/9
Test 35	1/3/8	2/6/11	2/6/10	1/5/10	2/5/10	2/7/11	3/9/14	2/7/10
Test 36	22/27/32	25/32/39	25/30/36	24/30/34	24/29/34	29/36/43	31/39/48	30/38/45
Test 37	29/33/45	34/41/54	35/40/52	33/38/47	33/39/49	38/45/57	40/47/63	35/45/54
Test 38	33/40/47	40/51/57	38/47/54	35/43/49	35/42/48	39/49/55	42/50/59	40/49/60
Test 39	37/45/52	45/53/60	44/54/59	38/48/54	40/47/56	44/55/61	46/58/69	43/56/62
Test 40	44/49/57	50/59/66	53/60/68	44/50/59	46/52/61	55/64/73	54/68/78	49/59/70

**Table 4:** Results of the hypothesise.

Test Case		ACOE	ACOS	ACOD	ACON	ACOU	EEMDE	PS	GSABC
Test 1	ACOE	–	0.438	0.402	0.443	0.500	0.385	0.419	0.440
	ACOS	0.568	–	0.496	0.536	0.568	0.423	0.439	0.560
	ACOD	0.598	0.504	–	0.573	0.598	0.434	0.503	0.569
	ACON	0.557	0.464	0.427	–	0.557	0.401	0.435	0.494
	ACOU	0.500	0.432	0.402	0.443	–	0.385	0.419	0.440
	EEMDE	0.615	0.577	0.566	0.599	0.615	–	0.579	0.595
	PS	0.581	0.561	0.497	0.565	0.581	0.421	–	0.562
	GSABC	0.560	0.440	0.431	0.506	0.560	0.405	0.438	–
Test 2	ACOE	–	0.321	0.315	0.440	0.380	0.436	0.309	0.298
	ACOS	0.679	–	0.480	0.624	0.604	0.610	0.472	0.465
	ACOD	0.685	0.520	–	0.638	0.609	0.617	0.477	0.470
	ACON	0.560	0.376	0.362	–	0.419	0.466	0.355	0.350
	ACOU	0.620	0.396	0.391	0.581	–	0.511	0.384	0.376
	EEMDE	0.564	0.390	0.383	0.534	0.489	–	0.375	0.369
	PS	0.691	0.528	0.527	0.645	0.616	0.625	–	0.481
	GSABC	0.702	0.535	0.530	0.650	0.624	0.631	0.519	–
Test 3	ACOE	–	0.303	0.347	0.398	0.465	0.067	7.890 × 10^−4^	0.187
	ACOS	0.697	–	0.580	0.589	0.598	0.214	0.177	0.323
	ACOD	0.653	0.420	–	0.508	0.531	0.151	0.104	0.278
	ACON	0.602	0.411	0.492	–	0.517	0.143	0.097	0.271
	ACOU	0.535	0.402	0.469	0.483	–	0.128	0.088	0.255
	EEMDE	0.933	0.786	0.849	0.857	0.878	–	0.378	0.667
	PS	1	0.823	0.896	0.903	0.912	0.222	–	0.791
	GSABC	0.813	0.677	0.722	0.729	0.745	0.333	0.209	–
Test 4	ACOE	–	0.005	0.244	0.240	0.330	0.103	0.045	0.309
	ACOS	0.995	–	0.874	0.865	0.945	0.708	0.665	0.901
	ACOD	0.756	0.126	–	0.487	0.711	0.288	0.279	0.663
	ACON	0.760	0.135	0.513	–	0.720	0.296	0.290	0.669
	ACOU	0.670	0.055	0.289	0.280	–	0.195	0.102	0.389
	EEMDE	0.897	0.292	0.712	0.704	0.805	–	0.388	0.789
	PS	0.955	0.335	0.721	0.710	0.898	0.612	–	0.833
	GSABC	0.691	0.099	0.337	0.331	0.611	0.211	0.167	–
Test 5	ACOE	–	0.209	0.201	0.400	0.353	0.348	0.122	0.341
	ACOS	0.791	–	0.458	0.681	0.620	0.612	0.366	0.605
	ACOD	0.799	0.542	–	0.688	0.623	0.618	0.397	0.610
	ACON	0.600	0.319	0.312	–	0.476	0.470	0.209	0.465
	ACOU	0.647	0.380	0.377	0.524	–	0.495	0.298	0.491
	EEMDE	0.652	0.388	0.382	0.530	0.505	–	0.312	0.498
	PS	0.878	0.644	0.603	0.791	0.702	0.668	–	0.679
	GSABC	0.659	0.395	0.390	0.535	0.509	0.508	0.321	–
Test 6	ACOE	–	0.038	7.765 × 10^−5^	0.114	0.266	6.742 × 10^−4^	6.009 × 10^−4^	0.043
	ACOS	0.962	–	0.102	0.777	0.891	0.289	0.276	0.691
	ACOD	1	0.898	–	0.991	1	0.792	0.660	0.945
	ACON	0.886	0.223	0.009	–	0.768	0.067	0.059	0.290
	ACOU	0.734	0.109	8.789 × 10^−4^	0.232	–	0.009	0.007	0.176
	EEMDE	1	0.711	0.208	0.933	0.991	–	0.355	0.887
	PS	1	0.724	0.340	0.941	0.993	0.645	–	0.892
	GSABC	0.957	0.309	0.055	0.710	0.824	0.113	0.108	–
Test 7	ACOE	–	0.039	0.165	0.045	0.389	1.335 × 10^−4^	1.004 × 10^−6^	9.876 × 10^−4^
	ACOS	0.961	–	0.858	0.720	0.933	0.221	0.115	0.290
	ACOD	0.835	0.142	–	0.419	0.776	0.009	0.001	0.067
	ACON	0.955	0.280	0.581	–	0.895	0.113	0.062	0.182
	ACOU	0.611	0.067	0.224	0.105	–	8.884 × 10^−4^	1.453 × 10^−4^	0.008
	EEMDE	1	0.779	0.991	0.887	1	–	0.399	0.662
	PS	1	0.885	0.999	0.938	1	0.601	–	0.794
	GSABC	1	0.710	0.933	0.818	0.992	0.338	0.206	–
Test 8	ACOE	–	7.542 × 10^−4^	0.009	0.019	0.025	0.001	7.544 × 10^−8^	5.980 × 10^−6^
	ACOS	1	–	0.595	0.634	0.809	0.553	0.167	0.225
	ACOD	0.991	0.405	–	0.562	0.622	0.444	0.004	0.027
	ACON	0.981	0.366	0.432	–	0.560	0.408	6.669 × 10^−4^	0.004
	ACOU	0.975	0.191	0.388	0.440	–	0.208	7.664 × 10^−5^	6.659 × 10^−4^
	EEMDE	0.999	0.447	0.556	0.592	0.798	–	0.096	0.122
	PS	1	0.833	0.996	1	1	0.904	–	0.726
	GSABC	1	0.775	0.973	0.996	1	0.878	0.274	–
Test 9	ACOE	–	0.004	5.545 × 10^−7^	0.054	0.029	5.898 × 10^−8^	7.653 × 10^−9^	6.645 × 10^−6^
	ACOS	0.996	–	0.005	0.913	0.834	7.706 × 10^−5^	8.744 × 10^−6^	0.012
	ACOD	1	0.995	–	1	1	0.004	1.975 × 10^−4^	0.992
	ACON	0.946	0.087	4.655 × 10^−6^	–	0.355	2.670 × 10^−7^	7.980 × 10^−8^	7.707 × 10^−5^
	ACOU	0.971	0.166	1.542 × 10^−4^	0.645	–	9.994 × 10^−6^	1.325 × 10^−6^	9.966 × 10^−4^
	EEMDE	1	1	0.996	1	1	–	0.238	1
	PS	1	1	1	1	1	0.762	–	1
	GSABC	1	0.988	0.008	1	1	9.642 × 10^−4^	1.565 × 10^−4^	–
Test 10	ACOE	–	1.222 × 10^−5^	9.667 × 10^−5^	4.448 × 10^−4^	0.007	8.890 × 10^−8^	3.897 × 10^−10^	8.754 × 10^−7^
	ACOS	1	–	1	1	1	2.238 × 10^−5^	9.688 × 10^−7^	9.998 × 10^−5^
	ACOD	1	7.766 × 10^−4^	–	0.993	1	8.890 × 10^−6^	3.346 × 10^−7^	2.346 × 10^−5^
	ACON	1	9.986 × 10^−5^	0.007	–	0.995	1.565 × 10^−6^	8.853 × 10^−8^	8.785 × 10^−6^
	ACOU	0.993	1.867 × 10^−5^	8.855 × 10^−4^	0.005	–	9.909 × 10^−7^	6.678 × 10^−9^	3.332 × 10^−6^
	EEMDE	1	1	1	1	1	–	0.998	1
	PS	1	1	1	1	1	0.002	–	1
	GSABC	1	1	1	1	1	5.323 × 10^−4^	4.455 × 10^−5^	–
Test 11	ACOE	–	0.185	0.206	0.295	0.310	0.182	7.656 × 10^−5^	0.203
	ACOS	0.815	–	0.558	0.688	0.756	0.397	0.234	0.502
	ACOD	0.794	0.442	–	0.597	0.698	0.335	0.008	0.490
	ACON	0.705	0.312	0.403	–	0.603	0.306	2.276 × 10^−4^	0.391
	ACOU	0.690	0.244	0.302	0.397	–	0.239	8.645 × 10^−4^	0.295
	EEMDE	0.818	0.603	0.665	0.694	0.761	–	0.245	0.610
	PS	1	0.766	0.992	1	1	0.755	–	0.873
	GSABC	0.797	0.498	0.510	0.609	0.705	0.390	0.127	–
Test 12	ACOE	–	0.156	0.256	0.320	0.355	0.002	5.895 × 10^−4^	0.036
	ACOS	0.844	–	0.560	0.599	0.635	0.387	0.324	0.425
	ACOD	0.744	0.440	–	0.552	0.580	0.345	0.303	0.398
	ACON	0.680	0.401	0.448	–	0.511	0.297	0.276	0.345
	ACOU	0.645	0.365	0.420	0.489	–	0.189	0.180	0.267
	EEMDE	0.998	0.613	0.655	0.703	0.811	–	0.458	0.582
	PS	1	0.676	0.697	0.724	0.820	0.542	–	0.604
	GSABC	0.964	0.575	0.602	0.655	0.733	0.418	0.396	–
Test 13	ACOE	–	0.041	0.026	0.207	0.290	0.037	6.766 × 10^−4^	0.025
	ACOS	0.959	–	0.208	0.751	0.876	0.309	0.220	0.201
	ACOD	0.974	0.792	–	0.832	0.902	0.633	0.315	0.508
	ACON	0.793	0.249	0.168	–	0.699	0.213	0.043	0.164
	ACOU	0.710	0.124	0.098	0.301	–	0.117	1.006 × 10^−4^	0.095
	EEMDE	0.963	0.691	0.367	0.787	0.883	–	0.279	0.361
	PS	1	0.780	0.685	0.957	1	0.721	–	0.681
	GSABC	0.975	0.799	0.498	0.836	0.905	0.639	0.319	–
Test 14	ACOE	–	0.027	0.009	0.176	0.055	8.560 × 10^−4^	6.745 × 10^−5^	1.875 × 10^−4^
	ACOS	0.973	–	0.277	0.658	0.511	0.149	0.011	0.085
	ACOD	0.991	0.723	–	0.775	0.733	0.256	0.095	0.156
	ACON	0.824	0.342	0.225	–	0.421	0.067	7.790 × 10^−4^	0.006
	ACOU	0.945	0.489	0.267	0.579	–	0.144	0.009	0.078
	EEMDE	1	0.851	0.744	0.933	0.856	–	0.243	0.387
	PS	1	0.989	0.905	1	0.991	0.757	–	0.612
	GSABC	1	0.915	0.844	0.994	0.922	0.613	0.388	–
Test 15	ACOE	–	0.005	0.009	0.030	0.045	2.674 × 10^−4^	6.745 × 10^−5^	0.006
	ACOS	0.995	–	0.560	0.714	0.993	0.379	0.204	0.507
	ACOD	0.991	0.440	–	0.665	0.898	0.125	0.055	0.465
	ACON	0.970	0.286	0.335	–	0.614	0.048	6.443 × 10^−4^	0.298
	ACOU	0.955	0.007	0.102	0.386	–	7.888 × 10^−4^	1.999 × 10^−5^	0.008
	EEMDE	1	0.621	0.875	0.952	1	–	0.499	0.632
	PS	1	0.796	0.945	1	1	0.501	–	0.804
	GSABC	0.994	0.495	0.535	0.702	0.992	0.368	0.196	–
Test 16	ACOE	–	0.018	6.232 × 10^−8^	5.178 × 10^−7^	4.181 × 10^−6^	1.455 × 10^−9^	5.743 × 10^−10^	8.823 × 10^−9^
	ACOS	0.982	–	0.013	0.024	0.031	4.532 × 10^−6^	1.094 × 10^−7^	7.895 × 10^−6^
	ACOD	1	0.987	–	0.510	0.528	7.890 × 10^−4^	8.643 × 10^−5^	0.012
	ACON	1	0.976	0.490	–	0.615	1.658 × 10^−4^	1.005 × 10^−5^	7.666 × 10^−4^
	ACOU	1	0.969	0.472	0.385	–	1.005 × 10^−5^	8.865 × 10^−6^	3.077 × 10^−5^
	EEMDE	1	1	1	1	1	–	0.411	0.624
	PS	1	1	1	1	1	0.589	–	1
	GSABC	1	1	0.988	1	1	0.376	5.565 × 10^−4^	–
Test 17	ACOE	–	5.167 × 10^−8^	3.344 × 10^−8^	6.437 × 10^−5^	8.222 × 10^−4^	5.543 × 10^−9^	8.644 × 10^−11^	3.534 × 10^−10^
	ACOS	1	–	0.604	0.951	1	0.401	0.087	0.176
	ACOD	1	0.396	–	0.940	1	0.287	8.766 × 10^−4^	0.005
	ACON	1	0.049	0.060	–	0.998	0.202	1.678 × 10^−4^	7.748 × 10^−4^
	ACOU	1	7.892 × 10^−5^	3.156 × 10^−5^	0.002	–	2.453 × 10^−6^	1.870 × 10^−7^	7.655 × 10^−7^
	EEMDE	1	0.599	0.713	0.798	1	–	0.226	0.314
	PS	1	0.913	1	1	1	0.774	–	0.488
	GSABC	1	0.824	0.995	1	1	0.686	0.512	–
Test 18	ACOE	–	0.140	0.243	0.399	0.591	0.005	4.886 × 10^−4^	0.001
	ACOS	0.860	–	0.560	0.702	0.874	0.254	0.108	0.164
	ACOD	0.757	0.440	–	0.631	0.798	0.120	0.067	0.096
	ACON	0.601	0.298	0.369	–	0.613	0.057	0.006	0.012
	ACOU	0.409	0.126	0.202	0.387	–	9.653 × 10^−4^	1.654 × 10^−4^	8.953 × 10^−4^
	EEMDE	0.995	0.746	0.880	0.943	1	–	0.237	0.316
	PS	1	0.892	0.933	0.994	1	0.763	–	0.590
	GSABC	0.999	0.836	0.904	0.988	1	0.684	0.410	–
Test 19	ACOE	–	0.020	0.029	0.227	0.038	1.887 × 10^−5^	6.673 × 10^−7^	3.572 × 10^−5^
	ACOS	0.980	–	0.515	0.801	0.675	0.208	0.008	0.399
	ACOD	0.971	0.485	–	0.768	0.508	0.058	9.777 × 10^−4^	0.168
	ACON	0.773	0.199	0.232	–	0.435	6.330 × 10^−4^	8.545 × 10^−5^	0.008
	ACOU	0.962	0.325	0.402	0.565	–	9.565 × 10^−4^	2.446 × 10^−4^	0.043
	EEMDE	1	0.792	0.942	1	1	–	0.376	0.605
	PS	1	0.992	1	1	1	0.624	–	0.875
	GSABC	1	0.601	0.832	0.992	0.957	0.395	0.125	–
Test 20	ACOE	–	0.355	0.433	0.452	0.518	0.014	6.674 × 10^−4^	0.003
	ACOS	0.645	–	0.577	0.600	0.773	0.276	0.168	0.201
	ACOD	0.567	0.423	–	0.525	0.697	0.188	0.079	0.107
	ACON	0.548	0.400	0.475	–	0.640	0.098	0.007	0.056
	ACOU	0.482	0.227	0.303	0.360	–	0.005	8.775 × 10^−5^	7.653 × 10^−4^
	EEMDE	0.986	0.724	0.812	0.902	0.995	–	0.288	0.316
	PS	1	0.832	0.921	0.993	1	0.712	–	0.664
	GSABC	0.997	0.799	0.893	0.944	1	0.684	0.336	–
Test 21	ACOE	–	0.027	0.031	0.047	0.042	7.534 × 10^−4^	5.909 × 10^−6^	1.166 × 10^−5^
	ACOS	0.973	–	0.599	0.868	0.772	0.245	0.023	0.086
	ACOD	0.969	0.401	–	0.763	0.648	0.196	0.002	0.011
	ACON	0.953	0.132	0.237	–	0.336	0.055	9.922 × 10^−5^	4.542 × 10^−4^
	ACOU	0.958	0.228	0.352	0.664	–	0.105	1.005 × 10^−4^	9.965 × 10^−4^
	EEMDE	1	0.755	0.804	0.945	0.895	–	0.198	0.344
	PS	1	0.977	0.998	1	1	0.802	–	0.602
	GSABC	1	0.914	0.989	1	1	0.656	0.398	–
Test 22	ACOE	–	0.034	0.021	0.037	0.028	1.301 × 10^−5^	6.446 × 10^−6^	5.655 × 10^−5^
	ACOS	0.966	–	0.206	0.697	0.390	0.134	0.054	0.237
	ACOD	0.979	0.794	–	0.875	0.630	0.303	0.256	0.379
	ACON	0.963	0.303	0.125	–	0.134	1.050 × 10^−4^	0.005	8.659 × 10^−4^
	ACOU	0.972	0.610	0.370	0.866	–	0.201	0.118	0.298
	EEMDE	1	0.866	0.697	1	0.799	–	0.406	0.611
	PS	1	0.946	0.744	0.995	0.892	0.594	–	0.689
	GSABC	1	0.763	0.621	1	0.702	0.389	0.311	–
Test 23	ACOE	–	5.127 × 10^−7^	3.654 × 10^−6^	6.008 × 10^−5^	5.945 × 10^−5^	1.334 × 10^−9^	8.644 × 10^−11^	6.523 × 10^−9^
	ACOS	1	–	0.630	0.883	0.752	0.002	7.674 × 10^−4^	0.008
	ACOD	1	0.370	–	0.765	0.611	1.004 × 10^−4^	6.653 × 10^−5^	8.653 × 10^−4^
	ACON	1	0.117	0.235	–	0.380	6.678 × 10^−8^	2.228 × 10^−9^	6.989 × 10^−7^
	ACOU	1	0.242	0.389	0.620	–	5.809 × 10^−6^	7.787 × 10^−7^	8.542 × 10^−5^
	EEMDE	1	1	1	1	1	–	0.249	0.562
	PS	1	1	1	1	1	0.751	–	0.957
	GSABC	1	1	1	1	1	0.438	0.043	–
Test 24	ACOE	–	9.878 × 10^−6^	2.289 × 10^−6^	8.254 × 10^−5^	2.634 × 10^−5^	1.034 × 10^−8^	7.653 × 10^−10^	8.777 × 10^−9^
	ACOS	1	–	0.635	0.951	1	1.556 × 10^−4^	2.786 × 10^−5^	1.002 × 10^−4^
	ACOD	1	0.375	–	0.870	0.966	8.323 × 10^−4^	5.670 × 10^−5^	9.997 × 10^−5^
	ACON	1	0.049	0.130	–	0.744	4.721 × 10^−5^	7.341 × 10^−6^	1.524 × 10^−5^
	ACOU	1	6.758 × 10^−4^	0.034	0.256	–	7.753 × 10^−6^	6.900 × 10^−8^	8.942 × 10^−7^
	EEMDE	1	1	1	1	1	–	7.773 × 10^−4^	0.005
	PS	1	1	1	1	1	1	–	0.628
	GSABC	1	1	1	1	1	0.995	0.372	–
Test 25	ACOE	–	3.657 × 10^−5^	0.037	0.046	4.453 × 10^−4^	3.652 × 10^−8^	5.653 × 10^−10^	7.890 × 10^−7^
	ACOS	1	–	0.892	0.991	0.655	7.674 × 10^−4^	8.342 × 10^−5^	0.309
	ACOD	0.963	0.108	–	0.567	0.189	8.650 × 10^−5^	9.765 × 10^−7^	9.564 × 10^−4^
	ACON	0.954	0.009	0.433	–	0.145	2.760 × 10^−5^	4.895 × 10^−7^	6.653 × 10^−4^
	ACOU	1	0.345	0.811	0.855	–	2.008 × 10^−4^	3.342 × 10^−5^	0.120
	EEMDE	1	1	1	1	1	–	0.305	0.904
	PS	1	1	1	1	1	0.695	–	1
	GSABC	1	0.691	1	1	0.880	0.096	3.342 × 10^−^^4^	–
Test 26	ACOE	–	7.620 × 10^−6^	3.986 × 10^−6^	1.876 × 10^−5^	0.041	7.843 × 10^−12^	7.780 × 10^−14^	6.742 × 10^−11^
	ACOS	1	–	0.622	0.953	1	1.980 × 10^−5^	5.432 × 10^−6^	9.431 × 10^−5^
	ACOD	1	0.378	–	0.969	1	7.532 × 10^−7^	8.854 × 10^−8^	4.562 × 10^−6^
	ACON	1	0.047	0.031	–	0.944	8.809 × 10^−8^	9.876 × 10^−10^	6.660 × 10^−7^
	ACOU	0.959	8.424 × 10^−4^	3.874 × 10^−4^	0.056	–	5.424 × 10^−10^	6.563 × 10^−12^	8.236 × 10^−9^
	EEMDE	1	1	1	1	1	–	0.317	1
	PS	1	1	1	1	1	0.683	–	1
	GSABC	1	1	1	1	1	4.523 × 10^−4^	6.531 × 10^−5^	–
Test 27	ACOE	–	2.848 × 10^−8^	4.012 × 10^−7^	4.645 × 10^−6^	8.834 × 10^−6^	3.653 × 10^−9^	1.009 × 10^−9^	2.123 × 10^−9^
	ACOS	1	–	0.608	0.654	0.875	1.753 × 10^−4^	9.784 × 10^−4^	1.109 × 10^−4^
	ACOD	1	0.392	–	0.568	0.835	4.642 × 10^−4^	2.006 × 10^−5^	1.653 × 10^−4^
	ACON	1	0.346	0.432	–	0.548	9.842 × 10^−5^	8.998 × 10^−6^	3.111 × 10^−4^
	ACOU	1	0.125	0.165	0.452	–	1.778 × 10^−5^	3.578 × 10^−7^	1.879 × 10^−5^
	EEMDE	1	1	1	1	1	–	0.014	0.231
	PS	1	1	1	1	1	0.986	–	0.527
	GSABC	1	1	1	1	1	0.769	0.473	–
Test 28	ACOE	–	2.006 × 10^−9^	6.955 × 10^−8^	1.664 × 10^−8^	5.115 × 10^−7^	1.892 × 10^−9^	1.754 × 10^−9^	1.056 × 10^−9^
	ACOS	1	–	0.597	1	1	0.104	0.003	0.078
	ACOD	1	0.403	–	1	1	5.670 × 10^−5^	2.085 × 10^−5^	7.753 × 10^−5^
	ACON	1	6.167 × 10^−4^	6.984 × 10^−4^	–	0.635	8.664 × 10^−6^	1.167 × 10^−6^	5.739 × 10^−6^
	ACOU	1	2.987 × 10^−4^	9.120 × 10^−4^	0.365	–	6.524 × 10^−7^	6.782 × 10^−8^	3.745 × 10^−7^
	EEMDE	1	0.896	1	1	1	–	0.512	0.595
	PS	1	0.997	1	1	1	0.488	–	0.410
	GSABC	1	0.922	1	1	1	0.405	0.590	–
Test 29	ACOE	–	4.675 × 10^−10^	3.043 × 10^−10^	5.783 × 10^−8^	3.665 × 10^−9^	1.524 × 10^−10^	6.785 × 10^−^^12^	7.543 × 10^−^^11^
	ACOS	1	–	0.388	1	1	5.623 × 10^−5^	4.563 × 10^−6^	1.245 × 10^−5^
	ACOD	1	0.612	–	1	1	7.905 × 10^−5^	9.342 × 10^−6^	6.894 × 10^−5^
	ACON	1	8.644 × 10^−4^	1.226 × 10^−5^	–	0.596	7.543 × 10^−7^	1.671 × 10^−8^	8.990 × 10^−8^
	ACOU	1	9.890 × 10^−4^	5.187 × 10^−5^	0.404	–	9.532 × 10^−7^	6.872 × 10^−8^	9.689 × 10^−8^
	EEMDE	1	1	1	1	1	–	0.001	0.204
	PS	1	1	1	1	1	0.999	–	0.606
	GSABC	1	1	1	1	1	0.796	0.394	–
Test 30	ACOE	–	7.453 × 10^−10^	1.768 × 10^−10^	2.875 × 10^−9^	4.093 × 10^−9^	8.543 × 10^−13^	2.901 × 10^−13^	6.453 × 10^−11^
	ACOS	1	–	0.705	1	1	6.346 × 10^−7^	3.246 × 10^−7^	5.895 × 10^−6^
	ACOD	1	0.295	–	1	1	2.005 × 10^−7^	1.652 × 10^−7^	1.564 × 10^−6^
	ACON	1	7.463 × 10^−5^	3.658 × 10^−5^	–	0.811	6.897 × 10^−10^	3.455 × 10^−10^	7.090 × 10^−8^
	ACOU	1	8.156 × 10^−6^	3.652 × 10^−6^	0.189	–	5.675 × 10^−10^	1.400 × 10^−10^	5.763 × 10^−8^
	EEMDE	1	1	1	1	1	–	0.398	1
	PS	1	1	1	1	1	0.602	–	1
	GSABC	1	1	1	1	1	4.907 × 10^−4^	2.689 × 10^−4^	–
